# The complete chloroplast genome of sweet tea (*Lithocarpus polystachyus*)

**DOI:** 10.1080/23802359.2019.1638841

**Published:** 2019-07-13

**Authors:** Yueqiao Li, Wei Guo, Ping He, Longhua Yu

**Affiliations:** a Experimental Center of Subtropical Forestry, Chinese Academy of Forestry, Xinyu, P. R. China;; b Taishan Academy of Forestry Sciences, Taian, P. R. China

**Keywords:** *Lithocarpus polystachyus*, chloroplast genome, Illumina sequencing

## Abstract

*Lithocarpus polystachyus*, also known as the sweet tea, is a plant of the family Fagaceae. It is widely distributed in southern China, India, and Thailand. The chloroplast (cp) genome of *L. polystachyus* is 161,217 bp in size containing 122 unique genes, including eight rRNA genes, 37 tRNA genes, and 77 protein-coding genes (PCGs). Phylogenetic analysis exhibited that *L. polystachyus* was most related to *L. balansae*.

*Lithocarpus polystachyus*, also known as the sweet tea, is a plant of the family Fagaceae. It is an evergreen tree and reaches a height of 7–15 m. It grows in dense forests at altitudes over 400 m and is widely distributed in southern China, India and Thailand (Institute of Botany, Chinese Academy of Sciences 1994). Local people made beverage and traditional herbal medicine with *L. polystachyus* leaves showing anti-diabetic and anti-hypertensive biological activities (Hou et al. 2011, 2012). The genomic sequence information is urgently needed to promote molecular evolution, systematics research, conservation and utilization of *L. polystachyus*. The objectives of the present study were to reconstruct the cp genome of *L. polystachyus* and assess phylogenetic relationships.

Fresh young leaves were sampled from a 3-year-old *L. polystachyus* tree at Nanyuan, Binjiang, Yichun, Jiangxi, China (27.62°N, 114.58°E) and chilled with liquid nitrogen immediately. The voucher specimen (accession no. NY_20190305_YC_JXC) was stored at −80 °C in Experimental Center of Subtropical Forestry, Chinese Academy of Forestry. Genomic DNA (gDNA) was obtained from homogenized leaf tissues using a modified CTAB protocol (Doyle and Doyle 1987). The quantity and quality of the purified gDNA were detected by Nanodrop 8000 and via the Agilent 2100 Bioanalyzer. A library with 350 bp fragments inserted was constructed with 1 μg purified DNA and high-throughput sequenced with paired end (PE) reads of 2 × 150 bp on Illumina Hiseq 2500 platform. Raw reads were filtered and trimmed to remove low quality and contaminated reads by trim_galore v0.4.4. Total 8.9 Gb of clean data were aligned to the *Quercus tarokoensis* complete cp genome (GenBank no. MF135621) as a reference using bowtie2 v2.2.4 (Langmead and Salzberg 2012) and assembled with SPAdes v3.10.1 (Bankevich et al. 2012). The final cp genome was annotated using HMMER 3.1b2 (Finn et al. 2011), ARAGORN v1.2.38 (Laslett and Canback 2004), and DOGMA (Boore et al. 2004).

The cp genome of *L. polystachyus* (GenBank no. MK914534) is 161,217 bp in size with total AT content 63.3%. It contains a 18,968 bp small and 90,491 bp large single copy regions with AT contents 69.3% and 65.4%, respectively, and two 25,879 bp inverted repeat regions with AT content 57.3%. In the cp genome of *L. polystachyus,* there are 122 unique genes, including eight rRNA genes, 37 tRNA genes, and 77 PCGs. Fourteen genes, including ten PCGs (*rps7*, *ndhI*, *rpl2*, *ndhC*, *rpl20*, *rpoC2*, *rps19*, *rpl23*, *psbA*, and *atpA*) harbour one intron each, while the PCG *psaA* harbour two introns.

To perform the molecular phylogenetic analysis, 16 published complete cp genomes were aligned by MAFFT v7.307 (Katoh and Standley 2013). Finally, a maximum likelihood (ML) tree was constructed using RAxML v.7.2.6 with 1000 bootstraps under the GTRGAMMA model (Stamatakis 2006). The ML phylogenetic tree indicated that *L. polystachyus* was most related to *L. balansae* (Figure 1). Most nodes in the cp genome ML tree were strongly supported. 

**Figure 1.**
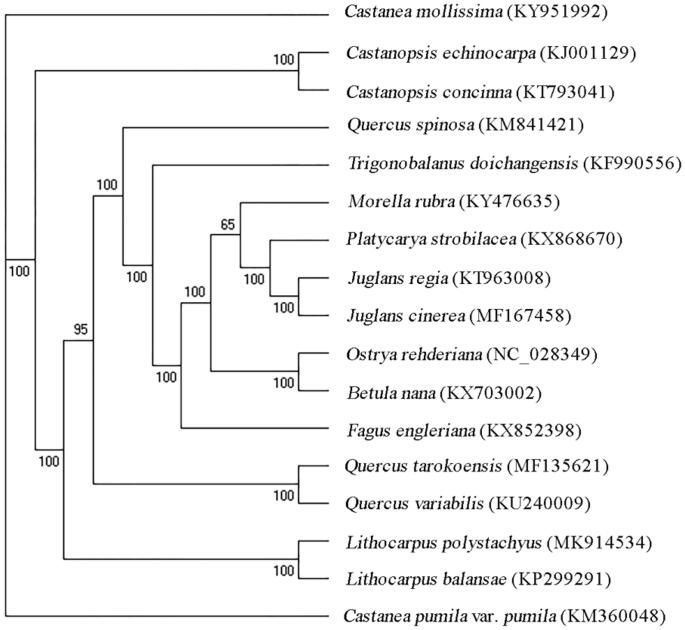
Phylogenetic tree based on 17 complete cp genome sequences. The bootstrap support values are shown next to the branches.
